# Progressive Adaptation of Subtype H6N1 Avian Influenza Virus in Taiwan Enhances Mammalian Infectivity, Pathogenicity, and Transmissibility

**DOI:** 10.3390/v17050733

**Published:** 2025-05-20

**Authors:** Zuoyi Zheng, Xifeng Chen, Rutian Zheng, Zhigang Yan, Long Li, Rirong Chen, Lifeng Li, Yongmei Liu, Yi Guan, Huachen Zhu

**Affiliations:** 1 Guangdong-Hong Kong Joint Laboratory of Emerging Infectious Diseases, Joint Laboratory for International Collaboration in Virology and Emerging Infectious Diseases (Key Laboratory of Ministry of Education), Joint Institute of Virology (Shantou University-The University of Hong Kong), Shantou University Medical College, Shantou University, Shantou 515063, China; 2 State Key Laboratory of Emerging Infectious Diseases (SKLEID), School of Public Health, Li Ka Shing Faculty of Medicine, The University of Hong Kong, Hong Kong SAR, China

**Keywords:** H6 subtype, zoonotic influenza, interspecies transmission, spillover, contact transmission, airborne transmission

## Abstract

The interspecies transmission of avian influenza viruses remains a significant public health concern. H6 viruses have gained attention following the first human infection by a chicken-origin H6N1 virus (A/Taiwan/02/2013, Hu/13), highlighting their zoonotic potential. To understand the evolutionary trajectory and mammalian adaptation of this Taiwan lineage, we compared two avian isolates (A/Chicken/Taiwan/CF19/2009, Ck/09; A/Chicken/Taiwan/2267/2012, Ck/12) and Hu/13 in vitro and in vivo. Hu/13 exhibited enhanced replication in MDCK cells, producing larger plaques and higher viral titers than Ck/09 and Ck/12. In BALB/c mice, Hu/13 demonstrated the highest pathogenicity and mortality, followed by Ck/12, while Ck/09 induced minimal morbidity. Hu/13 and Ck/12 replicated efficiently in respiratory tissues, eliciting robust cytokine responses and severe pulmonary lesions. In ferrets, Hu/13 showed relatively efficient transmission, infecting all direct physical-contact and two out of three airborne-contact ferrets, whereas Ck/09 failed to transmit. Histopathology confirmed escalating lung pathology from Ck/09 to Ck/12 and Hu/13. Whole-genome sequencing identified adaptive mutations in Hu/13 during ferret replication, though no canonical mammalian-adaptive changes (e.g., PB2-E627K or HA-Q226L) were detected. These findings demonstrate progressive mammalian adaptation, replication efficiency, and transmissibility within the Taiwan H6N1 lineage. Enhanced surveillance is crucial to monitor mammalian-adaptive mutations, informing pandemic preparedness and public health strategies.

## 1. Introduction

Avian influenza viruses (AIVs) represent a persistent global threat to public health and agriculture due to their capacity for cross-species transmission and pandemic potential. Over the past decades, China has witnessed the emergence of numerous AIV subtypes, including H3N8, H5Nx, H6N1, H7N9/H7N4, H9N2, and H10Nx [1]. Several outbreaks of avian-origin influenza in humans, often referred to as “bird flu”, have underscored the critical role of domestic birds, particularly chickens, in facilitating the spillover of novel avian subtypes into human populations [1,2,3,4].

Among the diverse subtypes of influenza A viruses, H6 viruses are particularly notable for their high prevalence in both wild and domestic avian species, and their establishment in terrestrial poultry [5,6,7,8]. Despite their widespread circulation, the zoonotic risk posed by H6 viruses has historically been underestimated. The first documented human infection with an avian-origin H6N1 virus, A/Taiwan/02/2013 (Hu/13), in 2013 marked a pivotal moment in understanding the evolutionary dynamics and interspecies spillover risks of H6 viruses. This case, involving a patient with no direct poultry exposure, demonstrated the potential for H6 viruses to infect humans without prior adaptation [9].

The subsequent isolation of a closely related H6N1 strain from a dog in 2014 further highlighted the adaptive capacity of this lineage [10,11], which had been enzootically established in Taiwanese poultry since 1997 [8,12], and is now expanding its host range in mammals [9,10]. Structural and genomic analyses have identified H6 viruses harboring mammalian-adaptive mutations in key functional domains [5,11,12,13,14,15,16,17,18], emphasizing the critical need to investigate their biological properties and zoonotic potential.

Previous studies on the dog-origin H6N1 isolate revealed that mutations such as PB2-E627K significantly enhance viral polymerase activity and pathogenicity in mice [11], highlighting the role of host adaptation in cross-species transmission. However, the evolutionary trajectory of the Taiwan H6N1 lineage, spanning avian-to-mammalian hosts, remains poorly characterized. To address this gap, we investigated three temporally distinct H6N1 strains: A/Chicken/Taiwan/CF19/2009 (Ck/09), A/Chicken/Taiwan/2267/2012 (Ck/12) [12], and Hu/13 [9], using different mammalian models. Comparative analysis of these isolates enables the identification of genetic and phenotypic changes that may enhance mammalian infectivity, pathogenicity, replication efficiency, and transmissibility.

Our findings demonstrate a progressive increase in mammalian adaptation within the Taiwan H6N1 lineage, with Hu/13 exhibiting enhanced replication efficiency, heightened pathogenicity in mice, and more efficient transmission in ferrets. These results provide critical insights into the evolutionary trajectory of H6 viruses and their potential to cause zoonotic infections, thereby informing surveillance and pandemic preparedness strategies. Furthermore, this study underscores the importance of continuous monitoring of H6 viruses for mammalian-adaptive mutations and highlights the need for sustained vigilance in understanding the factors that govern influenza virus evolution and cross-species transmission.

## 2. Materials and Methods

### 2.1. Ethical Compliance

All procedures involving animals were performed in accordance with institutional guidelines and internationally recognized standards for the humane treatment of laboratory animals. The experimental protocols were reviewed and approved by the Medical Animal Care and Welfare Committee of Shantou University Medical College (Approval No. SUMC2014-076) and the Committee on the Use of Live Animals in Teaching and Research (CULATR) of The University of Hong Kong (Approval No. 3265-14). Work with infectious agents was conducted in Biosafety Level 3 (BSL-3) containment facilities.

### 2.2. Virus Strains and Preparation

The human isolate A/Taiwan/02/2013 (Hu/13, EPI_ISL_143275) was isolated in Madin-Darby canine kidney (MDCK) cells and provided by Prof. Ming-Tsan Liu (Centers for Disease Control, Taiwan) [9]. The chicken strains, A/Chicken/Taiwan/CF19/2009 (Ck/09, EPI_ISL_139240) and A/Chicken/Taiwan/2267/2012 (Ck/12, EPI_ISL_158030), were isolated using 9- to 11-day-old embryonated chicken eggs and provided by Prof. Chwan-Chuen King (National Taiwan University) [12]. After plaque-purification, the viral stocks of these three viruses were prepared in MDCK cells (ATCC CCL-34), and titrated using hemagglutination tests, plaque assays, 50% tissue culture infectious dose (TCID_50_), and 50% egg infectious dose (EID_50_) methods [19,20]. Plaque morphology was quantified by measuring the diameters of 40 randomly selected plaques using a Zeiss Axiovert 40 inverted microscope (Carl Zeiss AG, Jena, Germany) at 50× magnification. Aliquots were stored at −80 °C until use.

### 2.3. Infection in Mice

Female Bagg Albino (BALB/c) mice (*Mus musculus*, 8–10 weeks old, specific-pathogen-free) were obtained from Hunan SJA Laboratory Animal Co. Ltd. (Changsha, China) and randomly allocated into experimental groups. Mice were anaesthetized via intramuscular injection of sodium pentobarbital (0.1 mg/g body weight; Sigma-Aldrich, St. Louis, MO, USA) and intranasally inoculated with 50 μL of phosphate buffered saline (PBS) containing serial dilutions (10^1^ to 10^6^ PFU, plaque-forming units) of the viruses (Ck/09, Ck/12, or Hu/13). Mock controls received PBS alone. Viruses with titers below 1 × 10^6^ PFU/50 μL were concentrated using 100 kDa molecular weight cut-off (MWCO) Amicon^®^ Ultra-0.5 centrifugal filter devices (Merck KGaA, Darmstadt, Germany).

At 4 days post-inoculation (dpi), three animals per dose group were euthanized for sample collection (nasal turbinate, trachea, lungs, and blood) by intramuscular administration of sodium pentobarbital (0.2 mg/g body weight). Tissues were processed for virological titration, histopathology, and gene expression analysis. Viral titers in the supernatant from 1 mL of PBS homogenates were determined by TCID_50_ assays in MDCK cells. The remaining mice (*n* = 5/group) were monitored daily for 14 days to assess survival and weight changes. Humane endpoints were defined as >25% weight loss. Mouse median lethal dose (MLD_50_) and 50% mouse infectious dose (MID_50_) were calculated using the Reed–Muench method [20].

### 2.4. Infection and Transmission in Ferrets

Male emasculated ferrets (*Mustela putorius furo*, 4–7 months old) were obtained from Wuxi Sangosho Biotechnology Co. Ltd. (Wuxi, China) and confirmed to be influenza-free and seronegative prior to use. Anesthesia was induced using a combination of Zoletil 50^®^ (Virbac, Carros, France) and Sumianxin II (Jilin Dunhua Shengda Animal Pharmaceutical Co., Ltd., Dunhua, China), administered intramuscularly as a compound anesthetic (0.25 mg Tiletamine + 0.25 mg Zolazepam + 5 mg Xylazine per kg body weight) prior to virus inoculation, serum collection, or intravenous drug administration in ferrets.

Nine ferrets per group were intranasally inoculated (IN) with 10^6^ PFU of virus (Ck/09, Ck/12, or Hu/13). At 1 dpi, these IN ferrets were transferred into three sets of transmission cages (i.e., 3 donors/cage), each housing a naïve ferret serving as the direct physical-contact (DC) animal. Additionally, for each set of IN and DC ferrets, another naïve ferret was placed in an adjacent cage (5 cm separation; inter-cage airflow <0.2 m/s) to serve as the airborne-contact (AC) ferret (Figure S1). Nasal washes and rectal swabs were collected daily for viral load quantification using TCID_50_. Three inoculated ferrets per group were euthanized at 4 and 7 dpi via intravenous injection of 100–150 mg/kg of pentobarbital solution for tissue collection. Surviving animals were humanely sacrificed at 14 dpi/dpc (days post-contact) for serum collection. Two ferrets inoculated with PBS were included as mock controls and sacrificed at 14 dpi for serum and tissue collection. None of the mock-infected ferrets were sacrificed at 4 and 7 dpi.

### 2.5. Hemagglutination Inhibition (HI) Test

Antibody titers in serum (14 dpi/dpc) were determined by the HI assay using 0.5% turkey erythrocytes, as previously described [19]. HI titers of 40 or above were defined as seroconverted following influenza virus infection.

### 2.6. Histopathological and Immunohistochemical (IHC) Studies

Tissues were fixed in 4% neutral-buffered formaldehyde for hematoxylin and eosin (HE) staining and IHC detection of viral nucleoprotein (NP), as previously described [21,22].

### 2.7. Cytokine/Chemokine Quantification

Total RNA was extracted from lung homogenates using the QIAamp Viral RNA Mini Kit (Qiagen, Valencia, CA, USA), treated with RNase-Free DNase (Qiagen), and reverse-transcribed into cDNA using the PrimeScript™ RT reagent Kit (Takara, Dalian, China). Transcript levels of each gene were quantified by SYBR Green-based real-time polymerase chain reaction (PCR) on a LightCycler^®^ 480 system (Roche Diagnostics, Basel, Switzerland) using validated primer sets (Tables S1 and S2) [23,24,25,26,27,28,29,30,31]. Target gene expression levels were normalized to the expression of the endogenous control gene glyceraldehyde-3-phosphate dehydrogenase (*GAPDH*) using the delta cycle threshold (Ct) formula: ΔCt = Ct _target gene_ − Ct _GAPDH_. Relative fold changes in cytokine/chemokine gene expression were calculated using the comparative 2^−ΔΔCt^ method, where ΔΔCt = ΔCt _target animal group_ − ΔCt _mock-infected group_. Fold changes were derived by raising 2 to the power of the negative ΔΔCt value.

### 2.8. Whole Genome Sequencing of the Virus

Viral RNA was extracted from inocula and ferret airway specimens using the QIAamp Viral RNA Mini Kit (Qiagen, Valencia, CA, USA) and whole-genome amplification of the influenza virus was performed as previously described [22,32]. Libraries were prepared with the Nextera XT DNA Library Prep Kit (Illumina, San Diego, CA, USA) and sequenced on an Illunima MiSeq benchtop sequencer (2 × 250 bp paired-end; Illumina). Raw reads were preprocessed using PRINSEQ [33] and assembled into contigs with the GS De Novo Assembler (version 2.6, Roche Applied Science, Indianapolis, IN, USA). Amino acid substitutions were annotated relative to reference genomes (EPI_ISL_139240, EPI_ISL_158030 and EPI_ISL_143275 for Ck/09, Ck/12 and Hu/13, respectively), with variant calling at a frequency ≥5% and minimum coverage depth of 100 reads.

### 2.9. Statistical Analysis

Statistical analyses were performed using GraphPad Prism 9.0.0 (GraphPad Software). Data are presented as mean ± standard deviation (SD). For comparisons across multiple groups, a one-way analysis of variance (ANOVA) was first conducted to assess overall differences. If the ANOVA reached statistical significance, post hoc pairwise comparisons (Ck/09 vs. Ck/12, Ck/09 vs. Hu/13, and Ck/12 vs. Hu/13) were performed using Tukey’s honestly significant difference (HSD) tests to control the family-wise error rate. Statistical significance was defined as * *p* < 0.05, ** *p* < 0.01, *** *p* < 0.001, **** *p* < 0.0001, and ns (not significant) for *p* ≥ 0.05.

## 3. Results

### 3.1. Viral Replication in MDCK Cells

**Figure 1:**
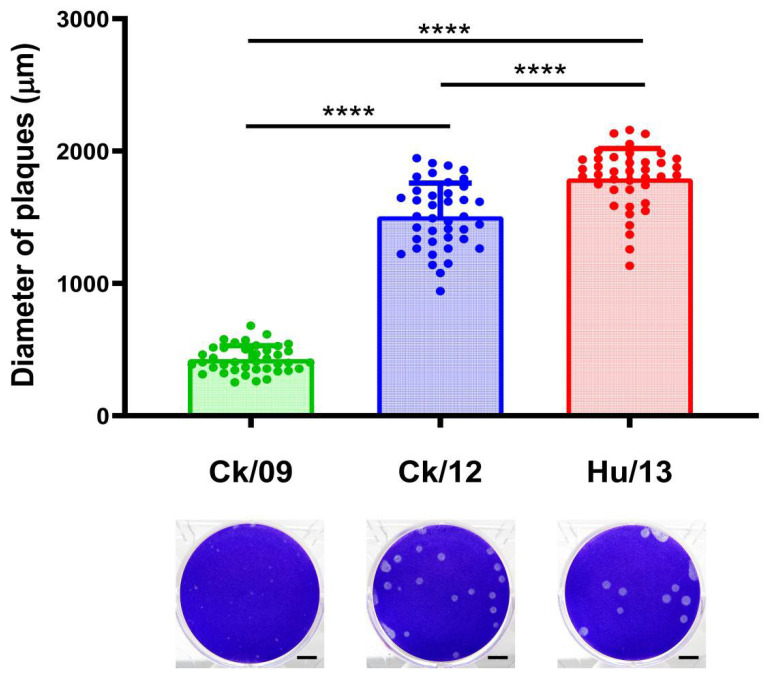
Plaques formed by the Taiwan H6N1 influenza viruses on MDCK cells. Mean plaque diameter for each virus (*n* = 40) is plotted above, error bars denote standard deviation (SD), **** indicates *p* < 0.0001. One-way ANOVA, followed by Tukey’s honestly significant difference (HSD) post hoc tests, was used for comparisons among the three viral groups. Plaque phenotypes are shown below; scale bars indicate 5 mm. Ck/09, A/Chicken/Taiwan/CF19/2009 (green); Ck/12, A/Chicken/Taiwan/2267/2012 (blue); Hu/13, A/Taiwan/02/2013 (red).

Comparative analysis of Ck/09, Ck/12, and Hu/13 revealed distinct replication phenotypes. The human isolate (Hu/13) exhibited significantly larger plaque diameters (1792 ± 227 μm) on MDCK cells compared to avian strains (Ck/09: 427 ± 102 μm; Ck/12: 1506 ± 251 μm; Figure 1). Viral titers quantified by EID_50_, TCID_50_, and PFU assays also demonstrated a progressive increase in replication efficiency: Ck/09 (EID_50_: 8.89 × 10^7^/mL; TCID_50_: 5.93 × 10^6^/mL; PFU: 2.25 × 10^6^/mL) < Ck/12 (EID_50_: 2.81 × 10^8^/mL; TCID_50_: 9.20 × 10^7^/mL; PFU: 6.00 × 10^7^/mL) < Hu/13 (EID_50_: 8.89 × 10^8^/mL; TCID_50_: 4.89 × 10^8^/mL; PFU: 3.00 × 10^8^/mL), with Hu/13 titers exceeding Ck/09 by 10-, 82.5-, and 133.3-fold, respectively (Table S3). These results suggest incremental adaptive mutations in the Taiwan H6N1 lineage enhancing mammalian cell tropism.

### 3.2. Viral Replication and Pathogenicity in BALB/c Mice

#### 3.2.1. Clinical Outcomes and Survival

**Figure 2:**
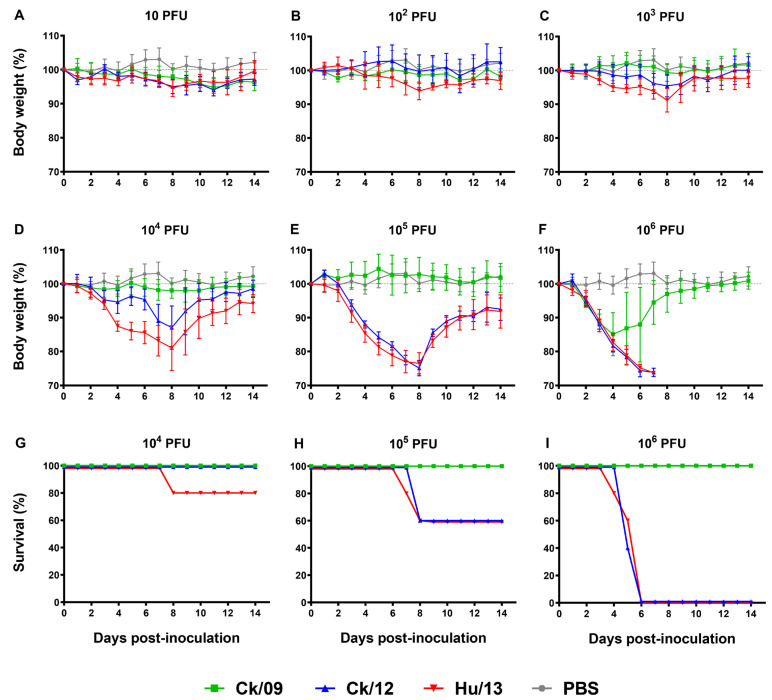
Clinical outcomes of Taiwan H6N1 influenza virus infection in mice. Groups of five BALB/c mice were inoculated with Ck/09 (green), Ck/12 (blue), or Hu/13 (red) at the indicated dose. Mock controls received phosphate buffered saline (PBS, gray) alone. Body weight changes (**A**–**F**) and survival rates (**G**–**I**) were monitored for 14 days post-inoculation (dpi). At the inoculation dose of 10–1000 PFU, no deaths were observed. Results are presented as mean ± SD. The horizontal dashed lines indicate baseline body weight on the inoculation day (100% body weight).

Mice inoculated with Hu/13 and Ck/12 exhibited dose-dependent morbidity, with 10^6^ PFU causing 100% mortality by 6 dpi and 60% survival at 10^5^ PFU, while Ck/09 showed minimal pathogenicity (100% survival at ≤10^6^ PFU; Figure 2G–I). MLD_50_ analysis further confirmed that Hu/13 (5.00 log_10_ PFU) and Ck/12 (5.17 log_10_ PFU) were more lethal than Ck/09 (≥6.50 log_10_ PFU; Table S4).

Weight loss mirrored survival trends: Hu/13 induced maximal weight reductions of over 25% (reaching the humane endpoint) at doses from 10^4^ to 10^6^ PFU, whereas Ck/09 caused ≤15.7% loss even at 10^6^ PFU (Figure 2D–F). The area under the curve (AUC) analysis of body weight changes revealed a significant reduction in Hu/13 compared to Ck/09 at doses ≥ 10^3^ PFU, and for Ck/12 compared to Ck/09 at doses ≥ 10^5^ PFU. A significant difference was also observed between Ck/12 and Hu/13 at 10^4^ PFU (one-way ANOVA followed by Tukey’s HSD tests, *p* < 0.05; Figure 2A–F).

#### 3.2.2. Viral Loads in Respiratory Tissues

**Figure 3:**
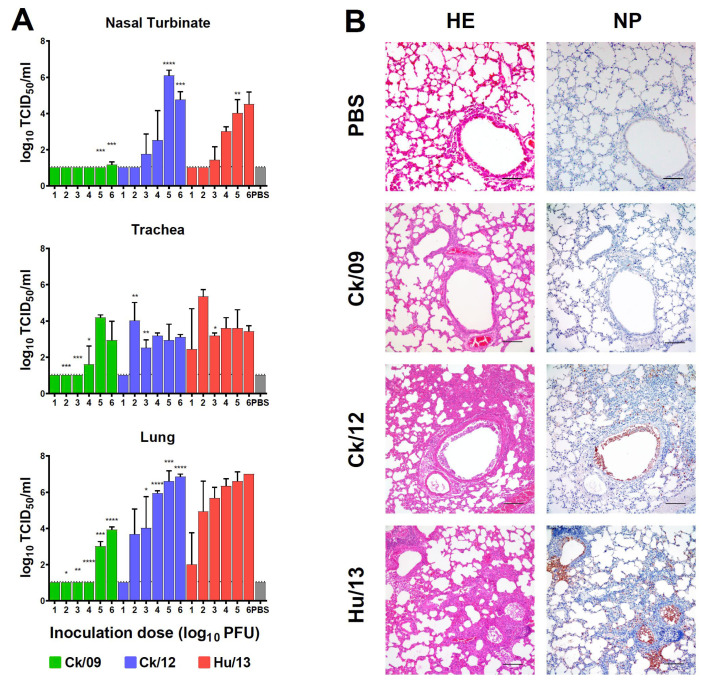
Replication of Taiwan H6N1 influenza viruses in the respiratory tissues of mice. Nasal turbinate, trachea, and lungs were harvested at 4 dpi (*n* = 3 per dose). (**A**) Viral titers were determined by TCID_50_ assays. Results are presented as mean ± SD. One-way ANOVA, followed by Tukey’s HSD post hoc tests, was used for comparisons among the three viral groups. Pairwise comparative results are presented as follows: comparisons between Ck/09 (green) and Hu/13 (red) are indicated above the Ck/09 groups; those between Ck/09 and Ck/12 (blue) are shown above the Ck/12 groups; and comparisons between Ck/12 and Hu/13 are labeled above the Hu/13 groups. No virus was detected in the mock-infected group inoculated with PBS (gray). The lower limit of detection is indicated by the horizontal dashed line. Statistical significance is denoted as * *p* < 0.05; ** *p* < 0.01; *** *p* < 0.001; **** *p* < 0.0001. (**B**) Histopathological and immunohistochemical (IHC) analyses were performed on lung tissues from mice inoculated with PBS or 10^5^ PFU of each virus. The left panel shows hematoxylin and eosin (HE) staining, while the right panel demonstrates viral nucleoprotein (NP) detection, with brown color indicating positively stained cells. Scale bars indicate 100 μm.

At 4 dpi, Hu/13 replicated efficiently across nasal turbinates (peak: 1.05 × 10^5^ TCID_50_/mL), trachea (5.93 × 10^5^ TCID_50_/mL), and lungs (1.05 × 10^7^ TCID_50_/mL). Ck/12 showed comparable replication in trachea (1.05 × 10^5^ TCID_50_/mL) and lung (1.87 × 10^7^ TCID_50_/mL), but higher replication in nasal turbinates at 10^5^ PFU (1.87 × 10^6^ TCID_50_/mL, *p* < 0.05). Ck/09 was restricted to trachea (1.87 × 10^4^ TCID_50_/mL) and lungs (1.05 × 10^4^ TCID_50_/mL), with significantly lower replication at most doses (Figure 3A). The minimal infectious doses for productive virus replication were highest for Ck/09 and lowest for Hu/13 (Figure 3A).

#### 3.2.3. Histopathology and Immune Responses

Immunohistochemistry revealed sparse NP-positive cells in Ck/09-infected lungs compared to dense foci in Ck/12- and Hu/13-infected tissues (Figure 3B, right panel). Histopathology confirmed escalating severity (Figure 3B, left panel) in Ck/12 and Hu/13, which showed severe pulmonary lesions, characterized by alveolar septal thickening and partial destruction of alveolar structures. Additionally, significant erythrocyte extravasation and inflammatory cell infiltration were evident within the alveoli and small bronchi, accompanied by necrosis and sloughing of epithelial cells in some small bronchioles. In contrast, Ck/09 infection resulted only in mild bronchial epithelial sloughing (Figure 3B, left panel).

**Figure 4:**
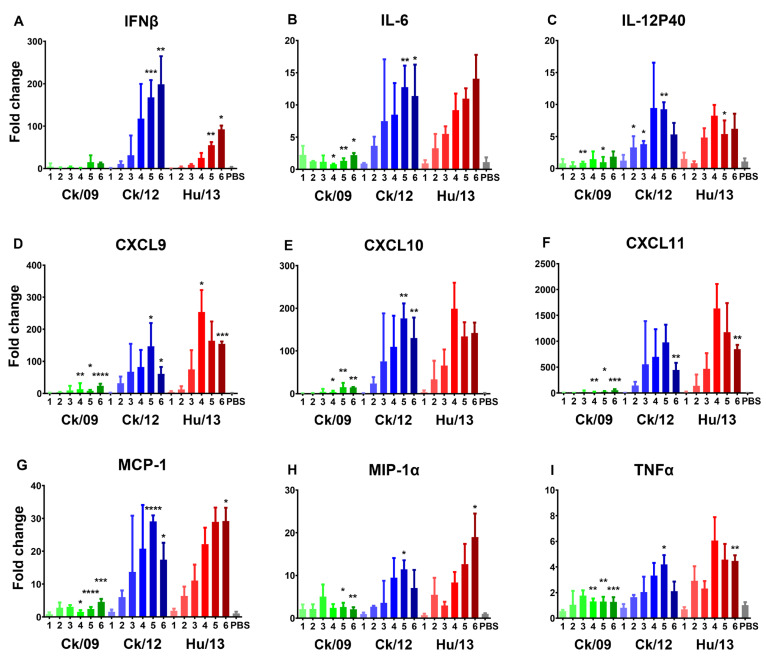
Transcriptional expression of cytokines/chemokines induced by the Taiwan H6N1 influenza viruses in the lungs of mice. Lung lobes of mice inoculated with PBS (gray), Ck/09 (green), Ck/12 (blue) or Hu/13 (red) were harvested at 4 dpi (*n* = 3 per inoculation dose). RNA expression levels of INFβ (**A**), IL-6 (**B**), IL-12P40 (**C**), CXCL9 (**D**), CXCL10 (**E**), CXCL11 (**F**), MCP-1 (**G**), MIP-1α (**H**), and TNFα (**I**) were measured using real-time PCR and normalized to GAPDH expression. Fold changes over the PBS inoculated group (mock controls) are shown, with the x-axis indicated as the inoculation doses (log_10_ PFU) of each virus. Results are presented as mean ± SD. One-way ANOVA, followed by Tukey’s HSD post hoc tests, was used for comparisons among the three viral groups. Pairwise comparisons between Ck/09 and Hu/13 are shown above the Ck/09 groups; those between Ck/09 and Ck/12 are indicated above the Ck/12 groups; and comparisons between Ck/12 and Hu/13 are labeled above the Hu/13 groups. Statistical significance is denoted as * *p* < 0.05; ** *p* < 0.01; *** *p* < 0.001; **** *p* < 0.0001.

Hu/13 and Ck/12 also triggered robust cytokine upregulation (Figure 4). Notably, Hu/13 induced significantly higher RNA levels of most tested cytokines/chemokines (IL-6, CXCL9, CXCL10, CXCL11, MCP-1, and TNFα) at doses ≥ 10^4^ PFU compared to Ck/09, while Ck/12 outcompeted Ck/09 in the expression of INFβ, IL-6, CXCL9, CXCL10, and MCP-1 at doses ≥ 10^5^ PFU (*p* < 0.05). However, Hu/13 induced lower levels of IFNβ than Ck/12 at doses ≥ 10^5^ PFU, but higher levels of CXCL9, CXCL11, MCP-1, MIP-1α, and TNFα than Ck/12 at 10^6^ PFU (*p* < 0.05).

#### 3.2.4. Seroconversion and Infectivity Parameters

Serological antibody analysis revealed distinct patterns of immune response among the three H6N1 virus strains in inoculated mice (Figure S2). Ck/09 required ≥10^4^ PFU for seroconversion (HI titers ≥ 40), with complete conversion at 10^6^ PFU. Ck/12 exhibited seroconversion at ≥10^2^ PFU, achieving full conversion at 10^4^ PFU, though mortality precluded antibody detection at higher doses. Hu/13 demonstrated the highest infectivity, with seroconversion at ≥10 PFU and complete conversion at 10^3^ PFU, despite mortality at elevated doses. The minimum infectious doses for seroconversion were 10^4^, 10^2^, and 10 PFU for Ck/09, Ck/12, and Hu/13, respectively, establishing an infectivity hierarchy of Ck/09 << Ck/12 < Hu/13 (Figure S2). Similarly, the MID_50_ values were also found to decrease sequentially: Ck/09 (4.50 log_10_ PFU) > Ck/12 (2.00 log_10_ PFU) > Hu/13 (1.50 log_10_ PFU; Table S4).

### 3.3. Viral Infection and Transmission Dynamics in Ferrets

#### 3.3.1. Clinical Observations

Ferrets intranasally inoculated with 10^6^ PFU of each H6N1 virus exhibited distinct clinical responses. Hu/13 caused the most pronounced effects, including significant temperature increases (up to 2 °C) and activity reduction for 1–2 days, along with marked sneezing and nasal discharge. Ck/12 induced moderate symptoms, while Ck/09 caused minimal changes, with less than 1 °C temperature increase and no inactivity. No body weight loss was observed in any infected ferrets.

#### 3.3.2. Viral Shedding and Transmission

**Figure 5:**
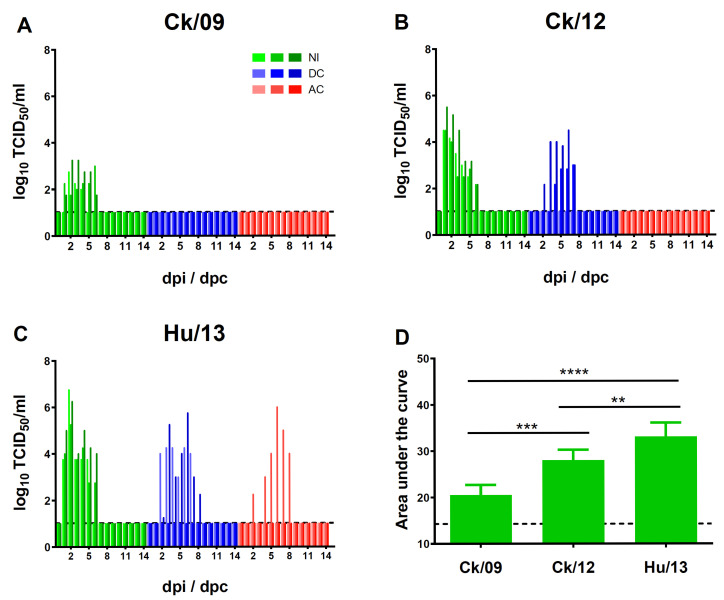
Infection and transmission of Taiwan H6N1 influenza viruses in ferrets. Groups of three ferrets were intranasally inoculated (IN, green) with 10^6^ PFU of Ck/09 (**A**), Ck/12 (**B**) or Hu/13 (**C**) and kept for 14 dpi. Additional six IN ferrets were sacrificed at 4 or 7 dpi (*n* = 3). For each group, three direct physical-contact (DC, blue) and three airborne-contact (AC, red) ferrets were introduced at 1 dpi. Virus shedding titers in nasal washes were monitored daily for 14 days post-inoculation or post-contact (dpi/dpc). The area under the curve (AUC) of viral shedding titers was calculated for each IN ferret and plotted (**D**). Results are presented as mean ± SD. The lower limit of detection is indicated by the horizontal dashed line. One-way ANOVA, followed by Tukey’s HSD post hoc tests, was used for comparisons among the three viral groups. Statistical significance is denoted as ** *p* < 0.01; *** *p* < 0.001; **** *p* < 0.0001.

**Figure 6:**
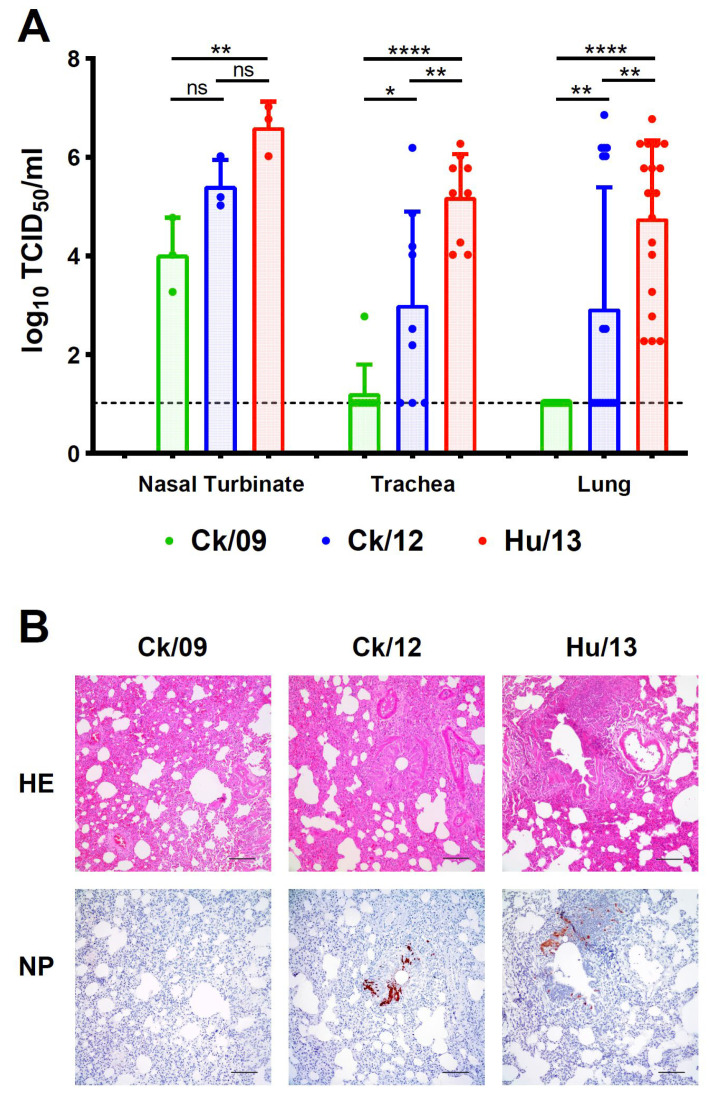
Replication of Taiwan H6N1 influenza viruses in the respiratory tissues of ferrets. Groups of three ferrets intranasally inoculated with 10^6^ PFU of Ck/09 (green), Ck/12 (blue) or Hu/13 (red) were sacrificed at 4 dpi. Viral titers in their nasal turbinate, trachea, and lungs were determined by TCID_50_ assays (**A**). Results are presented as mean ± SD. One-way ANOVA, followed by Tukey’s HSD post hoc tests, was used for comparisons among the three viral groups. Statistical significance is denoted as * *p* < 0.05; ** *p* < 0.01; **** *p* < 0.0001; ns (not significant) *p* ≥ 0.05. The lower limit of detection is indicated by the horizontal dashed line. Histopathological examination of lung tissues using HE staining and IHC analysis for viral NP detection (brown staining) are shown (**B**). Scale bars indicate 100 μm.

Hu/13 demonstrated efficient replication and transmission, with peak nasal wash titers reaching 5.93 × 10^6^ TCID_50_/mL. It infected all direct physical-contact ferrets (3/3) and two airborne-contact ferrets (2/3), leading to marked seroconversions. Ck/12 showed moderate transmission, infecting two DC ferrets (2/3) but none of the AC ferrets (0/3). Ck/09 failed to transmit to either DC or AC ferrets (Figure 5, Table 1). Viral loads in the respiratory tracts followed the hierarchy Hu/13 > Ck/12 > Ck/09 (Figure 6A). No viable virus was detected in rectal swabs throughout the experiment.

#### 3.3.3. Pathological Changes in Ferrets

Hu/13 induced severe pulmonary lesions, including bronchial necrosis and leukocyte infiltration, while Ck/09 caused mild pathology (Figure 6B). Ck/12 exhibited intermediate severity, with overt alveolar thickening and inflammatory cell infiltration (Figure 6B). IHC staining for viral NP antigens confirmed the higher replication of Hu/13 and Ck/12 in lung tissues compared to Ck/09 (Figure 6B).

#### 3.3.4. Seroconversion

All ferrets inoculated with Hu/13 seroconverted by 14 dpi, with HI titers ≥ 1280. Similarly, all Ck/12-inoculated ferrets seroconverted, with HI titers ranging from 640 to 1280. Ck/09-inoculated ferrets also seroconverted but with lower HI titers (320–640). Transmission groups mirrored these findings, with Hu/13 showing the highest seroconversion rates (Table 1). Notably, in the Hu/13 transmission group, one direct physical-contact ferret and one airborne-contact ferret showed seroconversion without detectable viral shedding (Figure 5C, Table 1).

#### 3.3.5. Proinflammatory Responses

**Figure 7:**
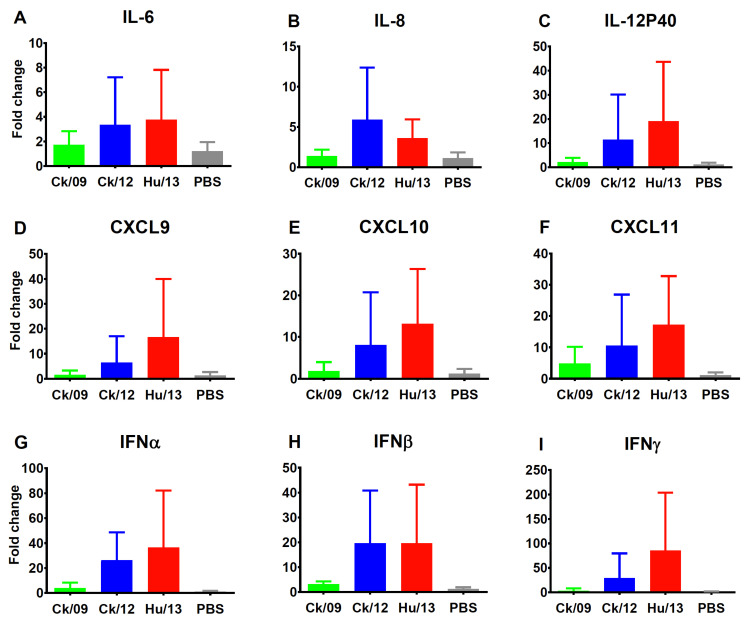
Transcriptional expression of cytokines/chemokines induced by the Taiwan H6N1 influenza viruses in the lungs of ferrets. Lung lobes from ferrets inoculated with PBS (gray), Ck/09 (green), Ck/12 (blue), and Hu/13 (red) were harvested at 4 dpi (*n* = 3 per group). RNA expression levels of IL-6 (**A**), IL-8 (**B**), IL-12P40 (**C**), CXCL9 (**D**), CXCL10 (**E**), CXCL11 (**F**), IFNα (**G**), INFβ (**H**), and IFNγ (**I**) were measured using real-time PCR and normalized to GAPDH expression. Fold changes over the PBS inoculated group (mock controls) are shown. Results are presented as mean ± SD. No statistically significant differences (*p* > 0.05) were observed using one-way ANOVA.

Proinflammatory cytokine and chemokine levels in lung tissues generally correlated with viral virulence. However, due to marked individual variations, differences among the virus groups were not statistically significant (*p* > 0.05, one-way ANOVA; Figure 7).

#### 3.3.6. Viral Mutations and Adaptation

Next-generation sequencing of Hu/13 during ferret passage identified a number of substitutions across the viral genome, with dominant mutations in PB1, HA, and NP. Notably, no canonical mammalian-adaptive mutations (e.g., PB2-E627K, PB2-D701N or HA-Q226L) were observed, suggesting that Hu/13 efficiently transmits in ferrets without requiring these well-characterized adaptive changes (Table S5).

## 4. Discussion

The interspecies transmission of AIVs continues to pose significant threats, particularly in densely populated regions with extensive poultry production, such as China. Among the most frequently detected influenza subtypes in aquatic birds, H6 viruses have sporadically spilled over into terrestrial poultry, leading to the establishment of enzootic clades [5,6,7,8]. The Taiwan H6N1 lineage exemplifies this evolutionary trajectory, having evolved into a distinct clade in chickens since 1997 and demonstrating increasing potential for mammalian adaptation over time [5,8,9,10,11,14]. This is evidenced by the first documented human and canine infections [9,10], as well as the progressive adaptation of temporally distinct strains in mammalian models, as highlighted in this study and previous research [8].

In this study, we compared the infectivity, pathogenicity, and transmissibility of three Taiwan H6N1 isolates (Ck/09, Ck/12, and Hu/13) in mammalian models. The results demonstrated a progressive enhancement in replication efficiency in MDCK cells, mice, and ferrets, with Hu/13 showing the highest capability, followed by Ck/12 and then Ck/09. Pathogenicity in mice and transmissibility in ferrets showed a similar trend, with Hu/13 exhibiting efficient contact transmission among ferrets. Notably, earlier H6N1 isolates from this Taiwan lineage, such as those from 1998 to 1999, were unable to replicate efficiently in mice, while isolates from 2002 to 2005 showed moderate replication [8]. In contrast, recent isolates like Ck/12 and Hu/13 displayed significantly enhanced replication in mammalian models, with Hu/13 causing severe morbidity and 100% mortality at 10^6^ PFU in mice. These findings suggest that prolonged circulation and adaptation of H6N1 viruses in chicken populations enhance their replication efficiency and transmissibility in mammals. This pattern mirrors observations in chicken-adapted H9N2 in China and its related derivative viruses (e.g., H3N8, H7N9, and H10N8), which have also demonstrated increasing zoonotic potential over time [3,4,22,34,35]. Furthermore, the replication capabilities of Ck/12 and Hu/13 in mice exceed those of H6 viruses isolated from other regions in China, as shown in comparative studies [7,36,37,38]. Collectively, these results underscore the growing zoonotic risk posed by Taiwan H6N1 viruses and highlight the need for continuous surveillance and research to mitigate public health threats.

Chickens play a critical role in the ecology of influenza viruses, serving as an intermediate host between aquatic birds and mammals [3,4,22,39,40]. The transition of H6 viruses from waterfowl to chickens has facilitated their adaptation to mammalian systems, as evidenced by the increasing replication efficiency and pathogenicity of recent Taiwan H6N1 isolates. The shift in viral replication sites from the intestines (in waterfowl) to the trachea (in chickens) has enabled H6 viruses to acquire α2–6 receptor binding capacity, a key step in mammalian adaptation [13,14,15,16,39]. This study provides further evidence that chickens act as a “bridge” for influenza viruses, promoting their evolution and potential spillover into humans and other mammals.

The enhanced zoonotic potential of chicken-adapted H6N1 may stem from multiple factors: (1) Receptor-binding shifts toward α2,6-linked sialic acids in chicken and mammalian respiratory tracts [39]. (2) Host immune and antiviral responses driving virus evolution, such as the lack of retinoic acid-inducible gene-I (RIG-I) in chickens, which orchestrates the production of antiviral interferon-stimulated genes (ISGs) [41]. (3) High evolutionary rates in chickens, which lack the evolutionary stasis seen in waterfowl reservoirs [6,40], promoting rapid adaptation. (4) Co-circulation of diverse subtypes (e.g., H5N2) enabling gene reassortment [12]. Chickens, as non-natural reservoir hosts, may also impose other unique selective pressures (e.g., body temperature, mucosal pH, enzymes) that inadvertently favor variants with broader host tropism.

The molecular basis for the H6 virus adaptation to mammals involves key mutations in multiple viral proteins. In the HA protein, E190V and G228S substitutions, prevalent post-2000 in Taiwan H6N1, enhance human-like α2,6-sialic acid affinity and bridging for avian-mammalian receptor tropism [5,9,13,14,15,16]. Compared to avian isolates, Hu/13 contains an additional P186L mutation (Table S6), which reduced its affinity for avian-type receptors [14]. Although the Taiwan H6N1 lineage retains HA-226Q, the triad of S137N, E190V, and G228S in many recent isolates may alleviate the requirement for a hydrophobic residue at the 226th position, increasing preference for human receptors [13]. In the NA protein, stalk deletions at positions 41–52 and 68–69 (N1-Δ14) of the Taiwan H6N1 lineage are associated with increased adaptation to terrestrial poultry and mammals [9,18,42], indicating their long-term adaptation in chickens and potential spillover to mammals.

Whole-genome comparative analysis revealed that Ck/09 had 134 and 126 amino acid differences with Ck/12 and Hu/13, respectively (Table S6). While Hu/13 lacks canonical mammalian-adaptive mutations like PB2-E627K, Q591R, and D701N in the internal genes [5,9], it exhibits other potential adaptive changes that may contribute to its enhanced replication and pathogenicity in mammals. These include consistent changes in Ck/12 and Hu/13, such as D249E, S265N, and I292V mutations in PB2 [43,44,45]; A85T, T97N, and L336M in PA [46,47,48,49]; and truncations in PB1-F2 (87 vs. 57 aa), a virulence modulator in mammals [50]. These findings suggest that H6 viruses can evolve to infect mammals through a combination of mutations, even in the absence of the most well-characterized adaptive markers. However, as the biological functions of these substitutions are primarily reported in H5N1, H1N1, or H7N9 strains [43,44,45,46,47,48,49,50], further investigation is needed to confirm their roles in the Taiwan H6N1 context.

While this study provides insights into the progressive evolution of H6N1 virus with increasing zoonotic potentials, several limitations must be acknowledged. First, the absence of post-2013 H6N1 isolates precludes the assessment of ongoing evolution in Taiwanese poultry, and the use of a limited number of strains may not fully recapitulate the genetic diversity and evolutionary dynamics of H6N1 in natural poultry populations. Second, while mouse and ferret models are widely used for influenza studies, they may not perfectly predict human infection, pathogenicity, and transmission patterns. Future research should incorporate more field isolates and explore alternative models to validate these findings. Third, while we identified amino acid variations between the avian and human isolates (Table S6) and adaptive mutations in ferret-passaged Hu/13 (Table S5), functional validation of these changes is needed. Additionally, investigating the role of host immune responses in shaping viral adaptation could provide a more comprehensive understanding of the factors driving cross-species transmission.

While both sexes are viable for viral infection studies [21,22,51], we used female mice and male emasculated ferrets to reduce the aggression and territorial behavior typical of intact males. This approach ensured social compatibility in group housing, minimized stress-related variability, and facilitated ease of handling. However, as gender, age and hormonal fluctuations may confound immune responses and viral transmission dynamics, the outcomes of H6N1 infection and transmission may vary among different animal models and populations [51].

Note that here we employed a 3:1:1 (IN:DC:AC) ratio for the ferret transmission study design (Figure S1), which differs from the strict 1:1 (donor:contact) or 1:1:1 (IN:DC:AC) ratios routinely used for airborne transmission assessment. The larger number of IN donors, combined with the use of DC ferrets, may have contributed to the enhanced transmission observed for the H6N1 isolates. Therefore, interpretation of the results and cross-study comparisons should be conducted with caution. However, for an influenza lineage that is enzootic in birds with limited mammalian spillover events and transmissibility, this non-conventional experimental setup and strategy may help us study these strains at a higher resolution. The increased number of donors allows us to identify subtle differences in transmission efficiency between human and avian isolates.

The progressive adaptation of H6N1 viruses in mammalian systems highlights the need for enhanced surveillance and control measures. The detection of subclinical infections in ferrets and the increasing replication efficiency of recent isolates, coupled with the silent persistence of H6N1 in Taiwan, underscore the potential for H6 viruses to cause outbreaks in humans. Given the high prevalence of H6 viruses in poultry and their ability to acquire mammalian-adaptive traits, continuous monitoring of viral evolution in poultry populations is essential. This includes tracking key mutations in HA, NA, and internal genes that may enhance viral replication, transmission, and pathogenicity in mammals. A One-Health approach and proactive risk assessment, including reverse genetics studies to pinpoint transmission determinants, are critical to mitigate pandemic threats.

## 5. Conclusions

The Taiwan H6N1 lineage provides a compelling example of the stepwise adaptation of avian influenza viruses to mammalian hosts, demonstrating how prolonged poultry adaptation can bridge avian-mammalian barriers. The enhanced replication efficiency, pathogenicity, and transmissibility of recent isolates, such as Hu/13, highlight the zoonotic potential of H6 viruses. These findings underscore the importance of ongoing surveillance and research to identify and mitigate the risks posed by H6 viruses, particularly in regions with extensive poultry production and human–animal contact. By understanding the molecular mechanisms of viral adaptation and the role of intermediate hosts like chickens, we can better prepare for and prevent future influenza pandemics.

## Figures and Tables

**Table 1:** Infection and transmission of the Taiwan H6N1 viruses in ferrets.

Ferret Groups	Virus	Shedding of Virus			Seroconversion	
		Animal Numbers	Peak Titers (log_10_TCID_50_/mL)	Duration (days)	Animal Numbers	HI Titers at 14 dpi/dpc
Intranasally inoculated (IN)	Ck/09	3/3	2.8, 3.0, 3.3	3, 6, 6	3/3	320, 640, 640
	Ck/12	3/3	4.5, 4.5, 5.5	5, 6, 6	3/3	640, 640, 1280
	Hu/13	3/3	5.3, 6.3, 6.8	5, 6, 6	3/3	1280, 1280, 1280
Direct physical-contact (DC)	Ck/09	0/3	-, -, -	-, -, -	0/3	-, -, -
	Ck/12	2/3	3.0, 4.5, -	4, 6, -	2/3	640, 640, -
	Hu/13	2/3	4.3, 5.8, -	6, 7, -	3/3	640, 1280, 1280
Airborne-contact (AC)	Ck/09	0/3	-, -, -	-, -, -	0/3	-, -, -
	Ck/12	0/3	-, -, -	-, -, -	0/3	-, -, -
	Hu/13	1/3	6.0, -, -	7, -, -	2/3	1280, 1280, -

-, Undetectable (virus titer < 10 TCID_50_/mL or HI titer < 10).

## Data Availability

The original contributions presented in this study are included in the article and Supplementary Material. Further inquiries can be directed to the corresponding authors.

## Supplementary Materials

The following supporting information can be downloaded at: https://www.mdpi.com/article/10.3390/v17050733/s1. Figure S1: Schematic diagram of the ferret transmission studies; Figure S2: Serological antibody titers in mice inoculated with Taiwan H6N1 influenza viruses. Table S1: Primers used for quantifying cytokine and chemokine expression in mice; Table S2: Primers used for quantifying cytokine and chemokine expression in ferrets; Table S3: Virus titers of the Taiwan H6N1 influenza viruses measured in MDCK cells and embryonated chicken eggs; Table S4: Mouse median lethal dose (MLD_50_) and 50% mouse infectious dose (MID_50_) of the Taiwan H6N1 influenza viruses; Table S5: Amino acid substitutions identified in the A/Taiwan/02/2013 (Hu/13) H6N1 influenza virus during infection and transmission in ferrets; Table S6. Amino acid variations identified in the three Taiwan H6N1 influenza virus isolates.

## Abbreviations

The following abbreviations are used in this manuscript:

AIV	avian influenza virus
Hu/13	A/Taiwan/02/2013
Ck/09	A/Chicken/Taiwan/CF19/2009
Ck/12	A/Chicken/Taiwan/2267/2012
CULATR	Committee on the Use of Live Animals in Teaching and Learning
BSL-3	Biosafety Level 3
MDCK	Madin-Darby canine kidney
TCID_50_	50% tissue culture infectious dose
EID_50_	50% egg infectious dose
BALB	Bagg Albino
PBS	phosphate buffered saline
PFU	plaque-forming unit
MWCO	molecular weight cut-off
dpi	day(s) post-inoculation
MLD_50_	mouse median lethal dose
MID_50_	50% mouse infectious dose
IN	intranasally inoculated
DC	direct physical-contact
AC	airborne-contact
dpc	day(s) post-contact
HI	hemagglutination inhibition
IHC	immunohistochemical
HE	hematoxylin and eosin
NP	nucleoprotein
PCR	polymerase chain reaction
GAPDH	glyceraldehyde-3-phosphate dehydrogenase
Ct	cycle threshold
SD	standard deviation
ANOVA	analysis of variance
HSD	honestly significant difference
ns	not significant
AUC	area under the curve
RIG-I	retinoic acid-inducible gene-I
ISGs	antiviral interferon-stimulated genes
CDC	Centers for Disease Control and Prevention
WHO	World Health Organization
