# Supplementary material for: Progressive Adaptation of Subtype H6N1 Avian Influenza Virus in Taiwan Enhances Mammalian Infectivity, Pathogenicity, and Transmissibility
Source: Viruses. 2025 May 20;17(5):733. doi: 10.3390/v17050733 (PMC12115762; doi:10.3390/v17050733)
Supplement: Supplementary file 1 [file viruses-17-00733-s001.zip › viruses-3629050-supplementary.pdf]

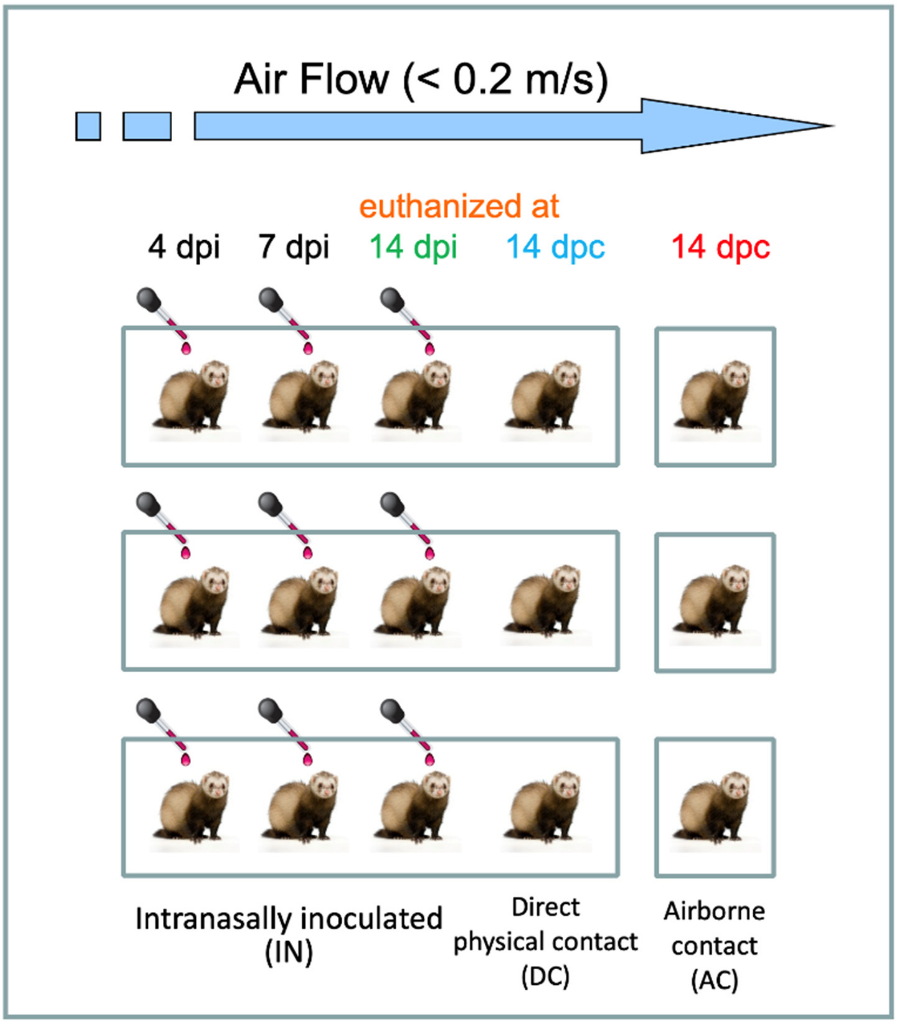

Figure S1. Schematic diagram of the ferret transmission studies. The diagram illustrates the experimental design for evaluating viral transmission in ferrets. For each virus strain tested, nine ferrets were intranasally inoculated (IN) with 10^6 PFU of virus. At 1 day post-inoculation (dpi), these IN ferrets were transferred into three sets of transmissions cages, each housing a naïve ferret serving as the direct physical-contact (DC) animal. Additionally, another naïve ferret was placed in an adjacent cage (5 cm separation; inter-cage airflow <0.2 m/s) to serve as the airborne-contact (AC) ferret. Nasal washes and rectal swabs were collected daily for viral titration. Three IN ferrets were euthanized at 4 and 7 dpi via intravenous injection of 100-150 mg/kg of pentobarbital solution for tissue collection. Surviving animals were humanely sacrificed at 14 dpi/dpc (days post-contact) for serum collection.

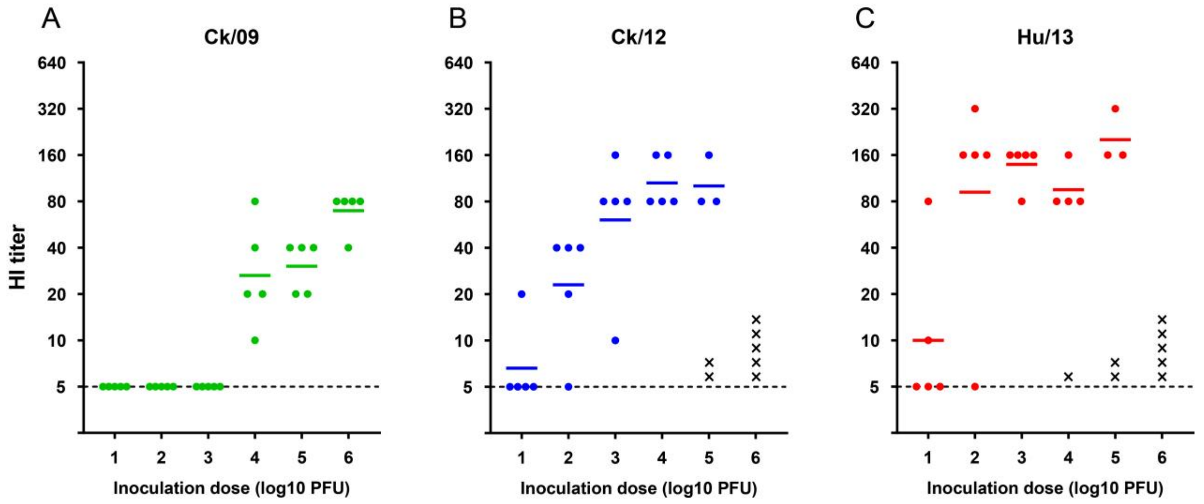

Figure S2. Serological antibody titers in mice inoculated with Taiwan H6N1 influenza viruses. (**A**) Ck/09, A/Chicken/Taiwan/CF19/2009 (green); (**B**) Ck/12, A/Chicken/Taiwan/2267/2012 (blue); (**C**) Hu/13, A/Taiwan/02/2013 (red). Blood samples were collected from mice (n=5 per group) inoculated with the indicated dose of each virus at 14 dpi. Antibody titers were measured using the hemagglutination inhibition (HI) test. The lower limit of detection is indicated by the horizontal dashed line. HI titers ⩾ 40 were defined as seroconverted following influenza virus infection. Mice that died during the experiment are marked with an "x." Each dot represents one mouse, with an HI titer of 5 indicating seronegativity, and the geometric mean is shown as a short vertical line.

Table S1. Primers used for quantifying cytokine and chemokine expression in mice.

Gene	Forward primer (5'→3')	Backward primer (5'→3')	Reference
Gapdh	TCACCACCATGGAGAAGGC	GCTAAGCAGTTGGTGGTGCA	[23]
Ifna1	GGACTTTGGATTCCCGCAGGAGAAG	GCTGCATCAGACAGCCTTGCAGGTC	[24]
Ifnb1	CCAGCTCCAAGAAAGGACGA	CGCCCTGTAGGTGAGGTTGAT	[25]
Ifng	TCAAGTGGCATAGATGTGGAAGAA	TGGCTCTGCAGGATTTTCATG	[23]
Il1b	TGTAATGAAAGACGGCACACC	TCTTCTTTGGGTATTGCTTGG	[24]
Il2	CCTGAGCAGGATGGAGAATTACA	TCCAGAACATGCCGCAGAG	[23]
Il6	GAGGATACCACTCCCAACAGACC	AAGTGCATCATCGTTGTTCATACA	[23]
Il10	GGTTGCCAAGCCTTATCGGA	ACCTGCTCCACTGCCTTGCT	[23]
Il12p40	GGAAGCACGGCAGCAGAATA	AACTTGAGGGAGAAGTAGGAATGG	[23]
Cxcl9	AGAACTCAGCTCTGCCATGAAGTC	CTAGGCAGGTTTGATCTCCGTTCT	[26]
Cxcl10	AGGGCCATAGGGAAGCTTGAAA	CGGATTCAGACATCTCTGCTCATC	[27]
Cxcl11	GGCTTCCTTATGTTCAAACAGGG	GCCGTTACTCGGGTAAATTACA	[28]
Mcp1	CTTCTGGGCCTGCTGTTCA	CCAGCCTACTCATTGGGATCA	[23]
Mip1a	ACCTGCTCAACATCATGAAGG	AGATGGAGCTATGCAGGTGG	[29]
Tnfa	CTGTAGCCCACGTCGTAGC	TTGAGATCCATGCCGTTG	[24]

Table S2. Primers used for quantifying cytokine and chemokine expression in ferrets.

Gene	Forward primer (5'→3')	Backward primer (5'→3')	Reference
GAPDH	AACATCATCCCTGCTTCCACTGGT	TGTTGAAGTCGCAGGAGACAACCT	[30]
IFNA1	ATGCTCCTGCGACAAATGAGGAGA	TTCTGCAGCTGCTTGCTGTCAAAC	[30]
IFNB1	GGTGTATCCTCCAAACTGCTCTCC	CACTCCACACTGCTGCTGCTTAG	[31]
IFNG	CCATCAAGGAAGACATGCTTGTCAGG	CTGGACCTGCAGATCATTCACAGGAA	[30]
IL2	TGCTGCTGGACTTACAGTTGCTCT	CAATTCTGTGGCCTTCTTGGGCAT	[30]
IL4	CGTTGAACATCCTCACAGCGAGAAAC	TTGCCATGTTCCTGAGGTTCCTGTGA	[30]
IL6	CAAATGTGAAGACAGCAAGGAGGCA	TCTGAAACTCCTGAAGACCGGTAGTG	[30]
IL8	AACCCACTCCACGCCTTTCCATC	GGCACACCTCTTTTCCATTGAC	[31]
IL10	TCCTTGCTGGAGGACTTTAAGGGT	TCCACCGCCTTGCTCTTATTCTCA	[30]
IL12P40	ATCGAGGTTGTGGTGGGTGCTATT	TAGGTTCATGGGTGGGTCTGGTTT	[30]

CXCL9	GGTGGTGTTCCTCTTTTGTTGA	GTTCCTTGGGTGGTGGTGAT	[31]
CXCL10	CCTGGCTTCACCGAGTTCT	AGTAGCAGCCCATGGAGTAAAA	[31]
CXCL11	CCTTCTTTCACATTCAGCGTTTCT	CTATAGCCATGCCCTTCACACTCA	[31]
TNFA	TGGAGCTGACAGACAACCAGCTAA	TGATGGTGTGGGTAAGGAGCACAT	[30]

Table S3. Virus titers of the Taiwan H6N1 influenza viruses measured in MDCK cells and embryonated chicken eggs.

Virus strain	Hemagglutination titer	EID_{50}/ml	$TCID_{50}$/ml	PFU/ml
A/Chicken/Taiwan/CF19/2009 (Ck/09)	256	8.89×10^{7}	5.93×10^{6}	2.25×10^{6}
A/Chicken/Taiwan/2267/2012 (Ck/12)	512	2.81×10^{8}	9.20×10^{7}	6.00×10^{7}
A/Taiwan/02/2013 (Hu/13)	512	8.89×10^{8}	4.89×10^{8}	3.00×10^{8}

Table S4. Mouse median lethal dose (MLD_{50}) and 50% mouse infectious dose (MID_{50}) of the Taiwan H6N1 influenza viruses.

Virus strain	MID_{50} (log_{10} PFU)	MLD_{50} (log_{10} PFU)
Ck/09	4.50	⩾ 6.50
Ck/12	2.00	5.17
Hu/13	1.50	5.00

Table S5. Amino acid substitutions identified in the A/Taiwan/02/2013 (Hu/13) H6N1 influenza virus during infection and transmission in ferrets.

Protein	Amino acid substitution	Inoculum	Intranasally inoculated ferret #1		Intranasally inoculated ferret #2		Intranasally inoculated ferret #3		Direct physical contact ferret #1	Direct physical contact ferret #3	Airborne contact ferret #2
			NT	Lung	NT	Lung	NT	Lung	NW	NW	NW
PB2	E520K	12.7	7.1	5.7	6.5	10.8	(4.2)	6.0	14.2	16.5	10.2
PB2	T521K	(1.4)	—	—	—	—	—	—	21.1	13.5	—
PB2	D671K	—	—	—	—	(1.7)	—	—	6.0	13.3	8.5
PB2	S714N	—	11.6	—	—	—	—	—	—	—	—
PB1	D175N	—	—	9.1	—	—	—	—	—	—	—
PB1	R260E	—	19.7	(4.6)	—	—	12.3	8.5	18	19.3	—
PB1	R260K	—	—	—	—	(1.1)	—	—	—	—	27.9

PB1	D305E	—	5.3	—	—	—	—	—	—	—	—
PB1	N312K	—	6.6	—	—	—	—	—	—	—	—
PB1	E383K	—	(2.6)	—	—	(2.9)	(1.7)	9.3	—	—	(3.9)
PB1	S384T	—	—	—	—	—	(1.8)	—	—	—	5.3
PB1	I459R	—	—	—	8.4	—	—	—	—	—	—
PB1	Q460K	—	(2.0)	—	11.7	(2.1)	(1.5)	(1.8)	—	—	(1.2)
PB1	N464D	—	7.3	—	—	(1.4)	—	—	5.2	(3.3)	—
PB1	Y689H	(3.6)	—	—	—	—	—	—	—	5.8	—
PA	T89M	—	—	—	—	—	(1.8)	76.7	—	—	—
PA	G235V	—	—	—	—	—	—	5.9	—	—	—
PA	P259T	—	—	—	—	5.7	—	—	—	—	—
PA	P295A	—	—	—	—	—	—	6.4	—	—	—
PA	K318R	—	—	—	—	—	—	8.4	—	—	—
PA	C489S	7.1	12.8	12.5	(4.7)	10.8	(2.6)	7.7	9.5	5.8	7.5
HA	V5I	(1.4)	72.7	81.2	72.1	65.7	77.0	71.3	82.5	84.7	77.9
HA	V6L	—	13.2	11.6	15.9	8.5	18.2	12.0	11.2	11.2	8.8
HA	D89|81N	—	—	32.3	—	—	—	—	—	—	—
HA	N149|137S	—	—	—	—	39.5	—	—	—	—	—
HA	T155|142A	—	—	44.2	—	37.5	—	—	—	—	—
HA	L200|186P	—	—	—	5.7	—	(2.5)	9.2	41.9	89.1	—
HA	L200|186I	—	—	—	—	—	—	14.1	—	—	—
HA	V204|190K	—	—	—	—	—	(2.6)	—	5.1	—	—
HA	N207|193K	98.4	69.2	78.3	90.7	86.2	84.9	86.5	35.1	26.4	85.2
NP	V186I	—	—	—	—	—	(1.2)	—	—	5.1	—
NP	V217I	—	—	—	—	—	(1.9)	(1.0)	10.4	15.5	—
NP	L313F	—	—	—	—	—	(1.3)	(1.4)	17.1	37.3	—
NP	S323A	—	—	—	—	—	(2.2)	(4.4)	14.6	37.1	—
NP	S359A	—	—	—	—	—	—	—	—	28.6	—
NP	I406V	—	—	—	—	—	(1.2)	—	—	19.9	—
NP	S482N	—	—	—	—	—	—	(3.0)	—	19.1	—
NA	Y156S	—	—	—	34.5	—	—	—	—	—	—
NA	S168N	—	—	6.7	—	—	—	—	—	—	—
M1	V213K	—	—	—	—	13.7	(1.4)	(4.9)	(5.1)	(2.1)	—
M1	V213T	—	—	—	—	—	—	—	—	5.8	—
M1	A215K	—	—	7.4	—	—	—	—	—	—	—
M2	I51T	—	11.1	—	—	—	—	—	—	—	—
NS1	L166M	—	—	—	—	—	5.1	—	—	—	—
NS2	E47K	—	—	—	5.7	(4.9)	—	—	5.2	6.8	7.1

Mutation rates (%) compared with the reference genome of A/Taiwan/02/2013 (Hu/13, EPI_ISL_143275) are shown in the table. Only positions with a substitution rate ≥ 5% are listed. NT, nasal turbinate; NW, nasal wash; —, substitution rates < 1%; ( ), mutation rates between 1% and 5%; red text, mutations rates > 25. For HA amino acid substitutions, the numbers following the vertical bar (|) correspond to the H3 numbering system.

Table S6. Amino acid variations identified in the three Taiwan H6N1 influenza virus isolates.

Protein	Position	Ck/09	Ck/12	Hu/13
PB2	24	T	N	T
	65	E	D	D
	102	N	S	S
	122	M	V	V
	156	E	A	A
	249	D	E	E
	251	R	R	K
	265	S	N	N
	292	I	V	V
	293	K	R	R
	315	M	I	M
	374	L	M	M
	411	V	I	I
	451	I	V	V
	453	P	T	T
	478	V	I	I
	555	R	K	R
	603	V	M	M
	648	P	L	L
	659	N	S	S
	676	A	V	V
	680	E	D	D
	718	R	K	K
PB1	52	K	N	N
	83	S	A	A
	111	I	M	M
	172	E	K	E
	195	M	M	T
	197	K	R	R
	200	I	V	V
	213	N	T	N
	261	N	S	S
	328	N	S	S
	375	S	S	N
	387	Q	K	K
	464	D	N	N
	566	S	T	T
	578	R	K	K
	579	L	M	M
	581	E	E	D
	695	I	L	L
	719	M	V	V
PB1-F2	21	K	T	T
	29	R	K	K

	36	I	T	T
	40	V	A	A
	43	L	P	P
	45	I	T	T
	58	W	*	*
PA	57	R	Q	Q
	59	E	D	E
	85	A	T	T
	86	I	M	M
	97	T	N	N
	100	V	I	V
	201	V	I	I
	206	D	E	E
	216	N	D	D
	262	R	K	K
	266	H	R	R
	325	P	S	P
	332	P	S	S
	336	L	M	M
	343	A	T	A
	364	S	N	S
	365	H	Q	Q
	432	V	V	I
	460	I	M	M
	565	V	M	V
	621	V	I	I
	626	K	R	R
PA-X	57	R	Q	Q
	59	E	D	E
	85	A	T	T
	86	I	M	M
	97	T	N	N
	100	V	I	V
	194	P	L	L
	206	I	K	K
	213	S	N	N
	236	S	L	S
	244	A	E	E
	250	Q	P	P
HA	5	I	V	V
	9	T	I	I
	18 | 12	R	K	K
	21 | 15	V	I	I
	46 | 40	I	V	V
	51 | 45	S	N	N
	55 | 49	N	K	K

	59 | 53	N	K	K
	69 | 62	R	K	K
	100 | 92	T	N	N
	114 | 105	V	L	L
	120 | 111	L	F	F
	122 | 113	G	E	G
	171 | 158	E	D	D
	174 | 160	A	T	T
	187 | 173	N	T	T
	200 | 186	P	P	L
	202 | 188	T	N	T
	214 | 200	R	K	K
	252 | 238	K	R	R
	287 | 271	N	D	D
	301 | 285	A	A	T
	329 | 313	S	N	S
	342 | 326	V	I	I
	343 | 327	E	A	A
	391 | 375	N	D	D
	402 | 386	T	N	N
	494 | 478	I	M	M
	509 | 493	D	K	K
	517 | 501	R	G	G
NP	186	I	V	V
	313	I	L	L
	318	S	S	P
	344	S	L	S
	373	T	A	T
NA	24	L	V	V
	26	I	V	V
	31	S	S	L
	34	I	I	V
	66	M	V	V
	74	I	N	N
	79	H	P	P
	149	M	V	V
	175	S	S	N
	192	L	M	L
	197	I	I	V
	227	V	I	V
	251	K	K	R
	350	T	S	T
	355	R	S	S
	375	V	V	M
	437	D	D	E

M1	14	I	I	V
	101	R	K	K
	125	A	S	S
M2	27	V	V	I
	51	V	I	I
	80	R	Q	Q
NS1	18	V	I	V
	27	L	R	L
	71	G	E	G
	86	A	T	N
	127	T	N	N
	137	V	I	I
	162	Q	P	P
	198	L	L	I
	205	S	S	N
	206	S	G	S
	211	R	G	G
	212	P	L	P
	213	P	S	P
	223	A	A	E
	226	I	V	V
	227	K	E	E
NS2	22	E	G	E
	48	A	A	T
	55	L	F	L
	85	H	Y	H

Residues that differ from the corresponding sites in the other two strains are highlighted in red text.

References

23. Giulietti, A.; Overbergh, L.; Valckx, D.; Decallonne, B.; Bouillon, R.; Mathieu, C. An overview of real-time quantitative PCR: applications to quantify cytokine gene expression. *Methods* **2001**, *25*, 386-401. http://doi.org/10.1006/meth.2001.1261.
24. Liu, G.; Friggeri, A.; Yang, Y.; Park, Y.J.; Tsuruta, Y.; Abraham, E. miR-147, a microRNA that is induced upon Toll-like receptor stimulation, regulates murine macrophage inflammatory responses. *Proc Natl Acad Sci U S A*. **2009**, *106*, 15819-15824. http://doi.org/10.1073/pnas.0901216106.
25. Martinez-Gil, L.; Goff, P.H.; Hai, R.; Garcia-Sastre, A.; Shaw, M.L.; Palese, P. A Sendai virus-derived RNA agonist of RIG-I as a virus vaccine adjuvant. *J Virol*. **2013**, *87*, 1290-1300. http://doi.org/10.1128/jvi.02338-12.
26. Liu, J.; Ma, X. Interferon regulatory factor 8 regulates RANTES gene transcription in cooperation with interferon regulatory factor-1, NF-kappaB, and PU.1. *J Biol Chem*. **2006**, *281*, 19188-19195. http://doi.org/10.1074/jbc.M602059200.
27. Lin, W.; Kemper, A.; McCarthy, K.D.; Pytel, P.; Wang, J.P.; Campbell, I.L.; Utset, M.F.; Popko, B. Interferon-gamma induced medulloblastoma in the developing cerebellum. *J Neurosci*. **2004**, *24*, 10074-10083. http://doi.org/10.1523/jneurosci.2604-04.2004.
28. Arima, Y.; Harada, M.; Kamimura, D.; Park, J.H.; Kawano, F.; Yull, F.E.; Kawamoto, T.; Iwakura, Y.; Betz, U.A.; Marquez, G.; et al. Regional neural activation defines a gateway for autoreactive T cells to cross the blood-brain barrier. *Cell* **2012**, *148*, 447-457. http://doi.org/10.1016/j.cell.2012.01.022.

29. Yoshida, S.; Yoshida, A.; Ishibashi, T.; Elner, S.G.; Elner, V.M. Role of MCP-1 and MIP-1alpha in retinal neovascularization during postischemic inflammation in a mouse model of retinal neovascularization. *J Leukoc Biol*. **2003**, *73*, 137-144. http://doi.org/10.1189/jlb.0302117.
30. Svitek, N.; von Messling, V. Early cytokine mRNA expression profiles predict Morbillivirus disease outcome in ferrets. *Virology* **2007**, *362*, 404-410. http://doi.org/10.1016/j.virol.2007.01.002.
31. Maines, T.R.; Belser, J.A.; Gustin, K.M.; van Hoeven, N.; Zeng, H.; Svitek, N.; von Messling, V.; Katz, J.M.; Tumpey, T.M. Local innate immune responses and influenza virus transmission and virulence in ferrets. *J Infect Dis*. **2012**, *205*, 474-485, http://doi.org/10.1093/infdis/jir768.
